# The clinical significance of preoperative plasma fibrinogen level and platelet count in resectable esophageal squamous cell carcinoma

**DOI:** 10.1186/s12957-015-0543-4

**Published:** 2015-04-22

**Authors:** Jianbo Wang, Hong Liu, Na Shao, Bingxu Tan, Qingxu Song, Yibin Jia, Yufeng Cheng

**Affiliations:** Department of Radiation, Qilu Hospital of Shandong University, 107 West Wenhua Road, Jinan, 250012 People’s Republic of China; Departments of Oncology, Shandong Provincial Hospital Affiliated to Shandong University, 324 Jingwu Weiqi Road, Jinan, People’s Republic of China

**Keywords:** Esophageal squamous cell carcinoma, Hyperfibrinogenemia, Thrombocytosis, Prognosis

## Abstract

**Background:**

Patients with malignant disease frequently present with activated coagulation pathways, which are potentially associated with tumor progression and prognosis. The aims of the study were to investigate the clinical significance of preoperative plasma fibrinogen level and platelet count in esophageal squamous cell carcinoma (ESCC) treated by curative surgery.

**Methods:**

A total of 119 patients with ESCC treated by curative surgery in Qilu Hospital of Shandong University were included in the study.

**Results:**

The preoperative plasma fibrinogen levels in the patients with ESCC ranged from 2.2 to 6.91 g/L (mean ± SD, 3.85 ± 0.95 g/L). The incidence of hyperfibrinogenemia was 43.7% (52/119, cut-off value 4.0 g/L). Hyperfibrinogenemia was found to be positively correlated with increased tumor length (*P* = 0.027), increased depth of invasion (*P* = 0.013), advanced pathological stages (*P* = 0.011), and disease recurrence (*P* = 0.026). The platelet counts ranged from 78 × 10^9^/L to 936 × 10^9^/L (mean ± SD, 254.51 ± 89.26 × 10^9^/L). The incidence of thrombocytosis was 20.2% (24/119, cut-off value 300 × 10^9^/L). Thrombocytosis was more frequently seen in male gender (*P* = 0.029) and non-smokers (*P* = 0.008). Plasma fibrinogen levels were significantly correlated with platelet counts (*r* = 0.018, *P* = 0.048). Hyperfibrinogenemia was significantly associated with poor disease-free (*P* = 0.009, hazard ratio (HR) = 1.784, 95% confidence interval (CI) = 1.153 to 2.761) and overall (*P* = 0.003, HR = 1.992, 95% CI = 1.259 to 3.152) survivals in univariate analysis, but not an independent prognostic indicator in multivariate analysis. Thrombocytosis was not significantly associated with disease-free (*P* = 0.765, HR = 0.918, 95% CI = 0.524 to 1.608) or overall (*P* = 0.809, HR = 1.072, 95% CI = 0.618 to 1.891) survivals in univariate analysis.

**Conclusions:**

The study suggested that hyperfibrinogenemia is a valuable predictor for disease progression in ESCC. Anticoagulation therapy might be considered to control cancer progression in future studies.

## Background

Esophageal cancer is the eighth most common cancer type and sixth leading cause of cancer death worldwide, which was responsible for 482,300 new cases and 406,800 deaths in 2008 [1]. In some high-risk regions such as North of China, esophageal cancer represents a major health problem, which is the fourth leading cause of cancer death. Squamous cell carcinoma is the major pathological type and accounts for more than 90% of esophageal cancer cases in Chinese patients [2]. Despite the advancement in diagnosis and treatment modalities, esophageal squamous cell carcinoma (ESCC) still shows a dismal prognosis with a 5-year survival rate less than 15% [3,4]. It is important to identify effective biomarkers to recognize unique biological characteristics of ESCC patients, in order to guide more individualized treatment.

It has been demonstrated that the alteration of coagulation pathways was associated with tumor progression and poor prognosis in various malignancies [5,6]. More than half of patients with metastatic disease have some abnormalities in hemostatic parameters [7]. Fibrinogen is a dimeric glycoprotein synthesized by hepatocytes, which plays a key role in clot formation and wound healing. It binds to platelets to support platelet aggregation after being converted to fibrin. Elevated plasma fibrinogen levels reflect a thrombophilic state that arises from the capacity of tumor cells to release coagulant molecules [8]. Fibrinogen is also a pro-inflammatory protein, which usually acts as an acute-phase protein in response to wound healing, infection, and inflammation [9]. Recently, the plasma fibrinogen level was found to correlate with clinicopathological factors and prognosis in gastric cancer, colon cancer, endometrial cancer, and so on [5,10,11]. Platelet is another crucial player of the coagulation system. Platelet could facilitate metastasis through promoting disseminated tumor cell survival in the circulation system, and extravasation and angiogenesis in the microenvironment of target sites [12]. Several studies have demonstrated that an elevated platelet count correlate with poor prognosis in several types of solid cancer, including lung cancer, colorectal cancer, gastric cancer, and so on [13-15]. However, the exact role of plasma fibrinogen level and platelet count in ESCC remains inconclusive.

In the present study, we examined the clinical significance of preoperative plasma fibrinogen level and platelet count in ESCC treated by curative surgery.

## Methods

### Patients

A total of 119 patients who underwent potential radical surgery for ESCC in Qilu Hospital of Shandong University between 1 January and 30 September 2008 were included in the study. Patients were already excluded because of palliative surgery, neoadjuvant treatment, perioperative mortality, distant metastasis, stage 0 disease, and lost to follow-up. The protocol of the study was approved by the Institutional Ethics Committee of the Qilu Hospital of Shandong University. All the patient demographic and clinical data including age, gender, histological grade, lymph node status, pTNM stage, and adjuvant treatment were abstracted from the clinical records. The stage of disease was determined according to the TNM system of the International Union Against Cancer (6th edition). A thorough histological examination was made using H&E-stained tissue preparations and the histological grade was determined according to the degree of differentiation of the tumor.

### Fibrinogen and platelet measurement

As a part of clinical routine, the pretreatment plasma fibrinogen levels and platelet counts were measured from early morning samples and were collected 24 h to 1 week before surgery after overnight fasting. Plasma fibrinogen levels and platelet counts greater than 4.0 g/L and 300 × 10^9^/L were defined as hyperfibrinogenemia and thrombocytosis, respectively, according to the normal reference range in our hospital.

### Follow up

Follow-up visit was performed every 3 months for the first 2 years and every 6 months for the next up to death or the end of the study. Data was censored at the last follow-up for patients without recurrence or death. At each visit, a clinical history was taken and a physical examination was performed. Routing diagnostic imaging methods included barium meal fluoroscopy and computer tomography. Disease-free survival (DFS) was defined from the date of the definitive surgery to the date of local or distant recurrence, mortality by any cause, or the last follow-up. Overall survival (OS) was calculated as the time from the date of surgery to that of mortality or censoring.

### Statistical analysis

Pretreatment plasma fibrinogen levels and platelet counts were reported as the mean ± SD. Continuous variables in different subgroups were compared using an unpaired *t*-test and one-way analysis of variance. Categorical variables were compared using chi-square test. Bivariate correlation analysis was performed using Pearson’s correlation coefficient analysis. Kaplan-Meier curves were used to estimate the distribution of DFS and OS, and log-rank test was performed to compare the difference between the survival curves. Variables, which were identified as statistically significant in univariate analysis, were included in the multivariate survival analysis using the Cox proportional hazard model. All statistical analyses were performed using SPSS 13.0 statistical software (SPSS Inc, Chicago, IL, USA). *P* values <0.05 were considered of statistical significance.

## Results

### Patient characteristics

Median age was 60 years (range, 42 to 78 years), and 80% of patients were male. Tumor locations were upper thoracic in 15 patients, mid-thoracic in 67 patients, and lower thoracic in 37 patients. The mean length of the tumor was 3.85 cm (range, 0.5 to 8.5). The histopathological differentiations were poor in 35 cases, moderate in 51 cases, and well in 33 cases. 75 patients (63%) had T3/T4 tumors. Forty-four patients (27%) have positive lymph nodes. The pathological stages were stage I in 21 patients, stage II in 58 patients and stage III in 40 patients. 71 patients (59.7%) received surgery alone, 11 (9.2%) received postoperative chemotherapy, 25 (21%) received postoperative radiotherapy, and 12 (10.1%) received postoperative chemoradiation. Eighty-one patients (68.1%) had recurrence, and 74 (62.2%) died during the follow-up. The estimated 1-, 3-, and 5-year DFS and OS rates were 75%, 49%, and 37% and 87%, 55%, and 41%, respectively. Median DFS was 33.8 months (range, 1.5 to 71.5 months). Median OS was 45.8 months (range, 2.6 to 71.5 months).

### Correlation between plasma fibrinogen, platelet count, and clinicopathological parameters

The plasma fibrinogen levels in preoperative esophageal cancer patients ranged from 2.2 to 6.91 g/L (mean ± SD 3.85 ± 0.95 g/L). The incidence of hyperfibrinogenemia was 43.7% (52/119, cut-off value 4.0 g/L). Hyperfibrinogenemia was found to be positively correlated with a larger tumor size (*P* = 0.027), increased depth of invasion (*P* = 0.013), advanced pathological stages (*P* = 0.011), and disease recurrence (*P* = 0.026). No significant correlation was identified between fibrinogen levels and tumor location or lymph node metastasis (Table 1).

**Table 1:** **Relationship of preoperative plasma fibrinogen level with clinicopathological parameter**

**Variable**	***N***	**Fibrinogen level**	***P*** **value**	**Fibrinogen level**		***P*** **value**
		**Mean ± SD**		**Normal**	**Hyperfibrinogenemia**	
		**(g/L)**		**(1.5 to 4 g/L)**	**(≥4 g/L)**	
Gender						
Male	95	3.94 ± 0.98	*0.034*	51	44	0.252
Female	24	3.48 ± 0.73		16	8	
Age						
<60	59	3.7 ± 0.94	0.101	38	21	0.077
≥60	60	3.99 ± 0.95		29	31	
Smoking history						
Yes	71	3.91 ± 0.95	0.371	29	19	0.457
No	48	3.75 ± 0.96		38	33	
Location						
Upper	15	3.56 ± 0.74	0.144	9	6	0.528
Middle	67	3.78 ± 0.99		40	27	
Lower	37	4.08 ± 0.93		18	19	
Tumor length						
<3.85 cm	64	3.56 ± 0.88	*<0.001*	42	22	*0.027*
≥3.85 cm	55	4.18 ± 0.93		25	30	
Differentiation						
Well	33	3.94 ± 0.81	0.782	17	16	0.676
Moderate	51	3.81 ± 1.09		31	20	
Poor	35	3.8 ± 0.87		19	16	
Tumor stage						
T1	18	3.25 ± 0.68	*0.001*	15	3	*0.013*
T2	26	3.67 ± 0.91		17	9	
T3	67	3.97 ± 0.92		33	34	
T4	8	4.69 ± 1.11		2	6	
Lymph node status						
Negative	75	3.77 ± 0.94	0.267	46	29	0.149
Positive	44	3.97 ± 0.97		21	23	
Pathological stages						
I	21	3.48 ± 0.81	*0.034*	15	6	*0.011*
II	58	3.79 ± 0.98		37	21	
III	40	4.12 ± 0.91		15	25	
Adjuvant treatment						
None	71	3.87 ± 0.95	0.962	37	34	0.307
Radiotherapy	25	3.83 ± 0.97		13	12	
Chemotherapy	11	3.9 ± 0.82		8	3	
CRT	12	3.72 ± 1.11		9	3	
Recurrence						
Yes	81	3.94 ± 0.97	0.116	40	41	*0.026*
No	38	3.65 ± 0.89		27	11	

SD, standard deviation; CRT, chemoradiotherapy. *P* < 0.05 is of significance.

The platelet counts ranged from 78 × 10^9^/L to 936 × 10^9^/L (mean ± SD 254.51 ± 89.26 × 10^9^/L). The incidence of thrombocytosis was 20.2% (24/119, cut-off value 300 × 10^9^/L). Thrombocytosis was more frequently seen in male gender (*P* = 0.029) and non-smokers (*P* = 0.008). There was no significant correlation between the platelet count and the larger tumor size, increased depth of invasion, advanced pathological stages, or disease recurrence (Table 2). A significant correlation between plasma fibrinogen levels and platelet counts was observed (*r* = 0.018, *P* = 0.048).

**Table 2:** **Relationship of preoperative platelet count with clinicopathological parameters**

**Variable**	***N***	**Platelet count**	***P*** **value**	**Platelet count**		***P*** **value**
		**Mean ± SD**		**Normal**	**Thrombocytosis**	
		**(×109/L)**		**<300 × 109/L**	**≥300 × 109/L**	
Gender						
Male	95	260.79 ± 96.13	0.127	72	23	*0.029*
Female	24	229.67 ± 47.93		23	1	
Age						
<60	59	254.86 ± 68.78	0.966	46	13	0.615
≥60	60	254.17 ± 106.22		49	11	
Smoking history						
Yes	71	267.39 ± 105.4	0.055	44	4	*0.008*
No	48	235.46 ± 53.23		51	20	
Location						
Upper	15	260.93 ± 82.51	0.696	12	3	0.964
Middle	67	248.36 ± 59.93		54	13	
Lower	37	263.05 ± 129.3		29	8	
Tumor length						
<3.85 cm	64	250.18 ± 103.94	0.571	54	10	0.183
≥3.85 cm	55	259.54 ± 68.96		41	14	
Differentiation						
Well	33	263.39 ± 136.03	0.793	26	7	0.568
Moderate	51	250.04 ± 69.68		39	12	
Poor	35	252.66 ± 54.62		30	5	
Tumor stage						
T1	18	207.56 ± 51.84	*0.034*	17	1	0.251
T2	26	247.58 ± 53.27		21	5	
T3	67	263.66 ± 105.27		52	15	
T4	8	306.13 ± 58.73		5	3	
Lymph node status						
Negative	75	250.89 ± 99.45	0.566	62	13	0.314
Positive	44	260.68 ± 69.14		33	11	
Pathological stages						
I	21	258.19 ± 168.65	0.233	17	4	0.636
II	58	246.48 ± 54.6		48	10	
III	40	264.23 ± 70.43		30	10	
Adjuvant treatment						
None	71	259.69 ± 105.06	0.823	56	15	0.788
Radiotherapy	25	253.48 ± 65.68		20	5	
Chemotherapy	11	235.55 ± 43.03		10	1	
CRT	12	243.42 ± 58.82		9	3	
Recurrence						
Yes		251.44 ± 58.06	0.675	66	15	0.513
No		261.05 ± 134.37		29	9	

SD, standard deviation; CRT, chemoradiotherapy. *P* < 0.05 is of significance.

### Survival analysis

**Figure 1:**
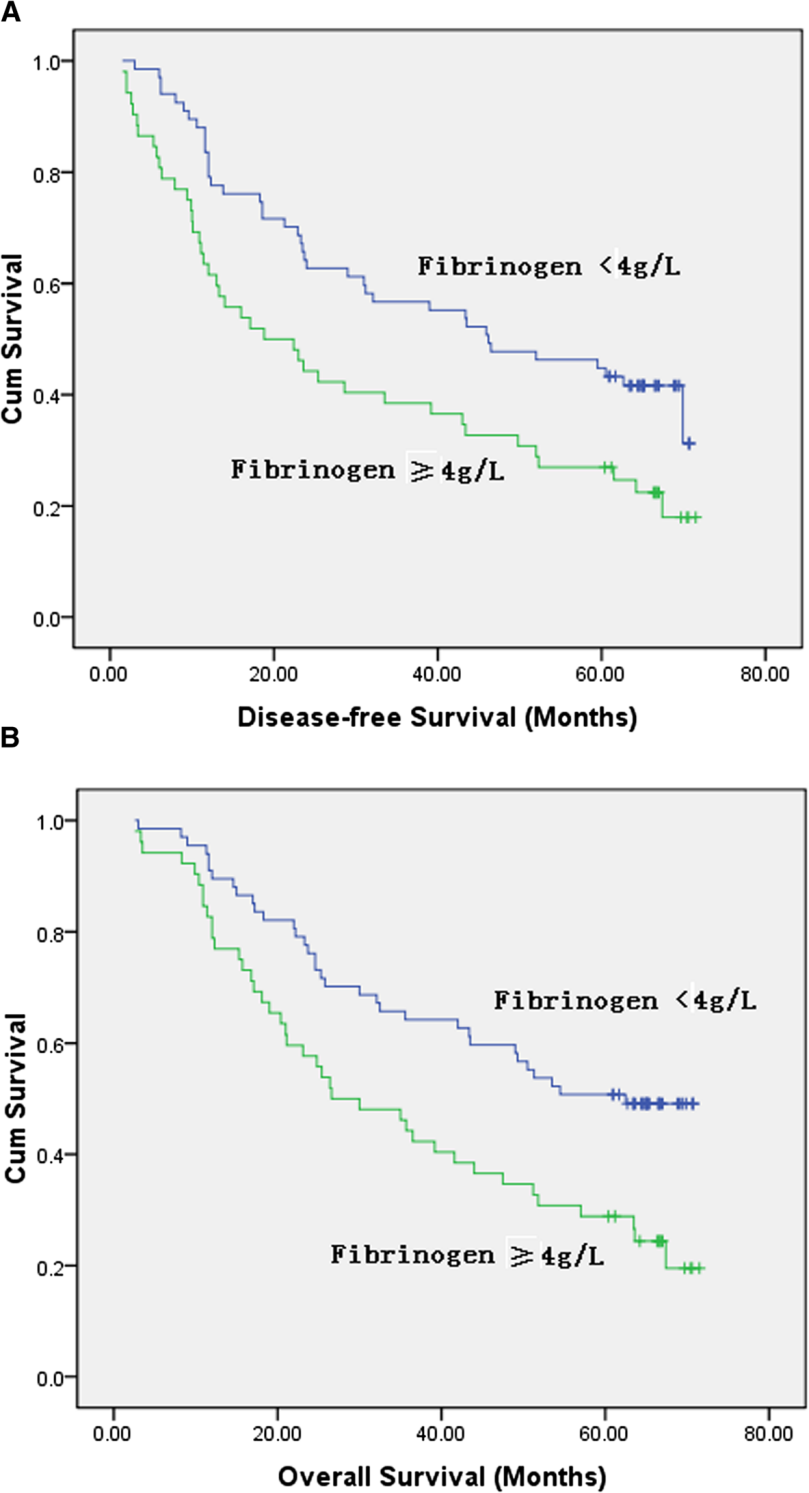
Kaplan-Meier analysis of preoperative plasma fibrinogen level in 119 patients with esophageal squamous cell carcinoma. Hyperfibrinogenemia were significantly associated with decreased disease-free survival **(A)** and overall survival **(B)**.

We assessed the prognostic values of the preoperative plasma fibrinogen level and platelet count in the ESCC patients included in our study, as shown in Table 3. Patients with hyperfibrinogenemia had lower 5-year disease-free survival (27% *vs*. 45%) and overall survival rates (29% *vs.* 51%) than those with normal plasma fibrinogen concentration. In univariate analysis, hyperfibrinogenemia was associated with poor disease-free (*P* = 0.009, hazard ratio (HR) = 1.784, 95% confidence interval (CI) = 1.153 to 2.761) and overall (*P* = 0.003, HR = 1.992, 95% CI = 1.259 to 3.152) survivals (Figure 1). Thrombocytosis was not correlated with disease survivals. Clinicopathological factors, which significantly associated with poor disease-free and overall survivals, were advanced T stages, lymph node metastasis, and advanced pTNM stages. In multivariate analysis, only pTNM stages were an independent predictor for disease-free survival (*P* < 0.001, HR = 2.985, 95% CI = 1.898 to 4.695), and both depth of invasion (*P* = 0.025, HR = 1.895, 95% CI = 1.084 to 3.313) and pTNM stages (*P* < 0.001, HR = 2.501, 95% CI = 1.52 to 4.114) were independent prognostic factors for overall survival.

**Table 3:** **Univariate analysis of survival of esophageal squamous cell carcinoma treated by curative surgery**

**Variable**	**Disease-free survival**	***P*** **value**	**Overall survival**	***P*** **value**
	**HR (95% CI)**		**HR (95% CI)**	
Gender (Female)	0.939(0.535 to 1.647)	0.826	0.937(0.523 to 1.677)	0.826
Age (≥60)	0.982(0.634 to 1.519)	0.933	1.09(0.69 to 1.721)	0.712
Smoking history	1.093(0.697 to 1.717)	0.698	1.139(0.712 to 1.825)	0.587
Location				
Upper	Ref.	0.432	Ref.	0.652
Middle	0.656(0.346 to 1.246)	0198	0.721(0.36 to 1.442)	0.355
Lower	0.694(0.349 to 1.378)	0.296	0.771(0.368 to 1.614)	0.49
Tumor length (≥3.85 cm)	1.178(0.761 to 1.824)	0.462	1.335(0.846 to 2.106)	0.215
Differentiation				
Well	Ref.	0.2	Ref.	0.141
Moderate	1.595(0.915 to 2.781)	0.1	1.69(0.931 to 3.067)	0.084
Poor	1.616(0.889 to 2.939)	0.116	1.819(0.965 to 3.427)	0.064
Tumor stage				
T1	Ref.	*0.011*	Ref.	*0.001*
T2	1.388(0.612 to 3.149)	0.432	1.591(0.605 to 4.188)	0.347
T3	2.295(1.117 to 4.713)	*0.024*	3.19(1.36 to 7.481)	*0.008*
T4	4.112(1.568 to 10.786)	*0.004*	5.968(2.058 to 17.301)	*0.001*
Lymph node metastasis	2.406(1.544 to 3.749)	*<0.001*	2.414(1.522 to 3.829)	*<0.001*
Pathological stages				
I	Ref.	*<0.001*	Ref.	*<0.001*
II	1.97(0.944 to 4.113)	0.071	2.561(1.069 to 6.133)	0.035
III	4.96(2.35 to 10.471)	*<0.001*	6.412(2.669 to 15.404)	*<0.001*
Adjuvant treatment				
None	Ref.	0.133	Ref.	0.073
Radiotherapy	1.672(0.984 to 2.839)	0.057	1.814(1.058 to 3.111)	*0.03*
Chemotherapy	0.732(0.312 to 1.714)	0.472	0.652(0.258 to 1.65)	0.367
CRT	1.549(0.756 to 3.174)	0.232	1.521(0.712 to 3.249)	0.279
Fibrinogen level (≥4 g/L)	1.784(1.153 to 2.761)	*0.009*	1.992(1.259 to 3.152)	*0.003*
Platelet count (≥300 × 10^9^ /L)	0.918(0.524 to 1.608)	0.765	1.072(0.618 to 1.891)	0.809

HR, hazard ratio; CI, confidence interval; CRT, chemoradiotherapy. *P* < 0.05 is of significance.

## Discussion

Recently, much attention has been paid to the relationship between hyperfibrinogenemia/thrombocytosis and malignant diseases. Elevated plasma fibrinogen levels and platelet counts have been found in some malignancies, such as gastric cancer, colorectal cancer, lung cancer, and so on [11,16-18]. In the present study, we analyzed the clinical significance of preoperative plasma fibrinogen level and platelet count in 119 patients with ESCC received curative surgery. We found that the incidence of hyperfibrinogenemia and thrombocytosis were 43.7% (52/119, cut-off value 4.0 g/L) and 20.2% (24/119, cut-off value 300 × 109/L), respectively, in the resectable ESCC patients. Hyperfibrinogenemia was found to be positively correlated with the increased tumor length, increased depth of invasion, and advanced pathological stages. Hyperfibrinogenemia was associated with poor disease-free and overall survivals in univariate analysis, but not an independent predictor for prognosis in multivariate analysis. Thrombocytosis was not associated with clinical outcome and clinicopathological parameters such as depth of invasion, lymph node metastasis, or pathological stages. We also observe a significant correlation plasma fibrinogen levels and platelet counts (*r* = 0.018, *P* = 0.048).

To our best knowledge, only two studies analyzed the clinical significance of plasma fibrinogen in ESCC. Takeuchi *et al*. [19] showed that pretreatment plasma fibrinogen correlates with tumor progression and metastasis in patients with ESCC. However, they did not show any association of pretreatment plasma fibrinogen with survivals in patients treated by surgery. Matsuda and colleagues [20] found that plasma fibrinogen level as a predictive marker for postoperative recurrence of ESCC in patients receiving neoadjuvant treatment. In the study, we firstly analyzed the clinical significance of pretreatment plasma fibrinogen in 119 ESCC treated by curative surgery without neoadjuvant treatment. Our study showed that hyperfibrinogenemia was significantly associated with advanced disease.

For thrombocytosis, the role of its prognostic significance is also inconclusive. Feng *et al*. [21] analyzed the clinical significance of preoperative thrombocytosis in a group of patients with ESCC and found that preoperative platelet count is a predictive factor for long-term survival in ESCC, especially in node-positive patients. Shimada *et al*. [22] found that a high platelet count is associated with tumor progression and poor survival in patients with esophageal cancer. However, the present study did not show that thrombocytosis was associated with clinical outcome and any pathological parameters in ESCC treat by curative surgery. The reason for the inconsistent results may be that our study included patients with relative early stage compared with previous studies.

The exact mechanism linking coagulation pathways and cancer progression remains unclear. Fibrinogen is a dimeric molecule with multiple integrin and non-integrin binding motifs, and cancer cells often express high levels of integrins or intercellular adhesion molecule 1. Fibrinogen deposition around tumor cells enhances the interaction between these cells and platelets, which effectively form microemboli in target organs [23]. Fibrinogen may also bind to growth factors, such as fibroblast growth factors and vascular endothelial cell growth factors, and thereby regulate endothelial cell proliferation and angiogenesis [24]. Fibrinogen layers help tumor cells block natural killer cytotoxicity with thrombin, which can protect tumor cells from the innate immune system [25].

Platelets, which were produced by mature bone marrow megakaryocytes, play an important role in arresting hemorrhage after tissue trauma or vascular injury. Platelets take an active part in the initiation and development of the inflammatory process by adhering to the cells of the vascular walls and by releasing of chemokines, cytokines, proteases, and procoagulants [26]. Platelets release various cytokines, including vascular endothelial growth factor (VEGF) and platelet-derived growth factor (PDGF), during blood clotting. The VEGF and PDGF family of proteins have a significant role in regulating tumor cell growth and angiogenesis [27]. Moreover, platelets expressing immunoregulatory protein such as glucocorticoid-induced TNF-related protein may protect the cancer cells from the host’s immune system [28].

The potential limitations of the present study include the use of a retrospective design. In order to select patients with a more uniform background, we only included esophageal cancer patients treated by potential curative surgery and excluded those received neoadjuvant treatment, which may also limit the general application of the results. Furthermore, larger prospective studies will be needed to confirm these preliminary results.

## Conclusions

Our study demonstrated that hyperfibrinogenemia is a valuable predictor for disease progression in ESCC. Anticoagulation therapy might be considered to control cancer progression in future studies. Additionally, the introduction of the convenient prognostic factors such as plasma fibrinogen can assist clinicians with better individualization of their therapeutic approach based on the risk stratification.

### Consent

Written informed consent was obtained from the patients for the publication of this report and any accompanying images.
